# Association between biochemical and hematologic factors with COVID-19 using data mining methods

**DOI:** 10.1186/s12879-023-08676-0

**Published:** 2023-12-21

**Authors:** Amin Mansoori, Nafiseh Hosseini, Hamideh Ghazizadeh, Malihe Aghasizadeh, Susan Drroudi, Toktam Sahranavard, Hanie Salmani Izadi, Amirhossein Amiriani, Ehsan Mosa Farkhani, Gordon A. Ferns, Majid Ghayour-Mobarhan, Mohsen Moohebati, Habibollah Esmaily

**Affiliations:** 1 International UNESCO Center for Health-Related Basic Sciences and Human Nutrition, Mashhad University of Medical Sciences, Mashhad, Iran; 2 Department of Applied Mathematics, Ferdowsi University of Mashhad, Mashhad, Iran; 3 Department of Biostatistics, School of Health, Mashhad University of Medical Sciences, Mashhad, Iran; 4 Faculty of Medicine, Islamic Azad University of Mashhad, Mashhad, Iran; 5 Division of Clinical Biochemistry, CALIPER Program, Pediatric Laboratory Medicine, the Hospital for Sick Children, Toronto, ON Canada; 6 Student Research Committee, Mashhad University of Medical Sciences, Mashhad, Iran; 7 Brighton & Sussex Medical School, Division of Medical Education, Falmer, Brighton, BN1 9PH Sussex UK; 8 Cardiovascular Research Center, School of Medicine, Mashhad University of Medical Sciences, Mashhad, Iran; 9 Social Determinants of Health Research Center, Mashhad University of Medical Sciences, Mashhad, Iran

**Keywords:** Data mining, Decision trees, SARS-COV-2, Biochemical, Hematologic, COVID-19

## Abstract

**Background and aim:**

Coronavirus disease (COVID-19) is an infectious disease that can spread very rapidly with important public health impacts. The prediction of the important factors related to the patient's infectious diseases is helpful to health care workers. The aim of this research was to select the critical feature of the relationship between demographic, biochemical, and hematological characteristics, in patients with and without COVID-19 infection.

**Method:**

A total of 13,170 participants in the age range of 35–65 years were recruited. Decision Tree (DT), Logistic Regression (LR), and Bootstrap Forest (BF) techniques were fitted into data. Three models were considered in this study, in model I, the biochemical features, in model II, the hematological features, and in model II, both biochemical and homological features were studied.

**Results:**

In Model I, the BF, DT, and LR algorithms identified creatine phosphokinase (CPK), blood urea nitrogen (BUN), fasting blood glucose (FBG), total bilirubin, body mass index (BMI), sex, and age, as important predictors for COVID-19. In Model II, our BF, DT, and LR algorithms identified BMI, sex, mean platelet volume (MPV), and age as important predictors. In Model III, our BF, DT, and LR algorithms identified CPK, BMI, MPV, BUN, FBG, sex, creatinine (Cr), age, and total bilirubin as important predictors.

**Conclusion:**

The proposed BF, DT, and LR models appear to be able to predict and classify infected and non-infected people based on CPK, BUN, BMI, MPV, FBG, Sex, Cr, and Age which had a high association with COVID-19.

## Introduction

The global numbers of new cases from Coronavirus Disease 2019 (COVID-19) continues to rise, the world’s agencies, institution and governments are still working towards identifying individuals who are at greatest risk of infectious [1]. Identification of these predictive factors will make it possible to optimized allocation the human and technical resources for management [2, 3]. In addition, such predictors would also allow designing the interventional studies to target patients at risk of worsening and progression to death [4].

Studies have shown that certain demographic factors are related to the severity of COVID-19 [2, 5, 6]. Among these, older age is an important predictor of mortality and male sex is a parameter in the proposed clinical severity risk scores [7]. Pre-existing conditions, such as diabetes mellitus, obesity, cardiovascular disease, hypertension (HTN), chronic lung diseases (particularly COPD), chronic kidney disease, immune-suppression and sickle cell disease, predispose patients to an adverse clinical course and elevated risk of intubation and death [8].

Regarding laboratory tests, studies have reported laboratory parameters that may predict COVID-19 prognosis [9]. Findings commonly in relation to poor outcomes including increased lactate dehydrogenase (LDH), C-reactive protein (CRP), D-dimer levels and high-sensitivity cardiac troponin I [10].

More knowledge of the specific symptoms and risk determinants of COVID-19 in different clinical settings are needed to properly treat these patients and to avoid disease complications [7, 11]. Thus, this study was conducted to assess and analyze treatment, laboratory and hospital results and the clinical and hematological features of COVID-19 patients at a Khorasan Razavi Health Center, Iran. The purpose of the current study was therefore to provide an overview of the relationship between COVID-19 and demographic, biochemical, and hematological features, in order to better understand the situation, improve the treatment and management of the disease in the future and present an image of the disease burden in Iran applying machine learning algorithms.

In many areas of medicine, machine learning techniques have been useful for prediction and classification. In machine learning, the two primary task categories are "supervised" and "unsupervised" [12]. An algorithm for supervised machine learning is a decision tree (DT) used in medical applications [13–16]. Traditional statistical techniques make it difficult to choose predictors, so we applied data mining techniques like DT to forecast the biochemical and hematologic measurements most closely associated with COVID-19. In the fields of medicine, public health, etc., logistic regression (LR) is applied to calculate the association between one or more independent (predictor) variables and a binary dependent (outcome) variable [17–19].

The Bootstrap Forest (BF) platform fits an ensemble model by averaging several DTs, each of which is fit to a bootstrap sample of the training data. Each split in each tree shows a random subset of the predictors.

## Materials and methods

### Study population

This study was conducted on a population of 13,170 in the age range of 35–65 years including 5780 subjects with severe acute respiratory syndrome coronavirus 2 (SARS-COV-2) and 7390 subjects without SARS-COV-2 from the MASHAD cohort study (Phase I) as previously described [20]. The Ethics Committee of the Mashhad University of Medical Sciences reviewed and approved the informed consent form, study protocol, and other study related documents. All participants provided informed, written consent.

### Blood sampling

According to a standard protocol, all blood samples were collected from an antecubital vein of all participants following 12–14 h of overnight fasting between 8–10 am in a sitting position. The details of laboratory measurements and cut-offs are explained in the baseline report of the MASHAD cohort study, as described previously [20].

### Demographic data

Health care professionals and a nurse gathered demographic characteristics (e. g. age, sex, and smoking status from participants by interviewing.

### Anthropometric assessments

Anthropometric measurements, including weight, height, body mass index (BMI) and waist circumference, were measured in all subjects of the research according to standardized protocols [20].

### Diagnosis of COVID-19

**Fig. 1:**
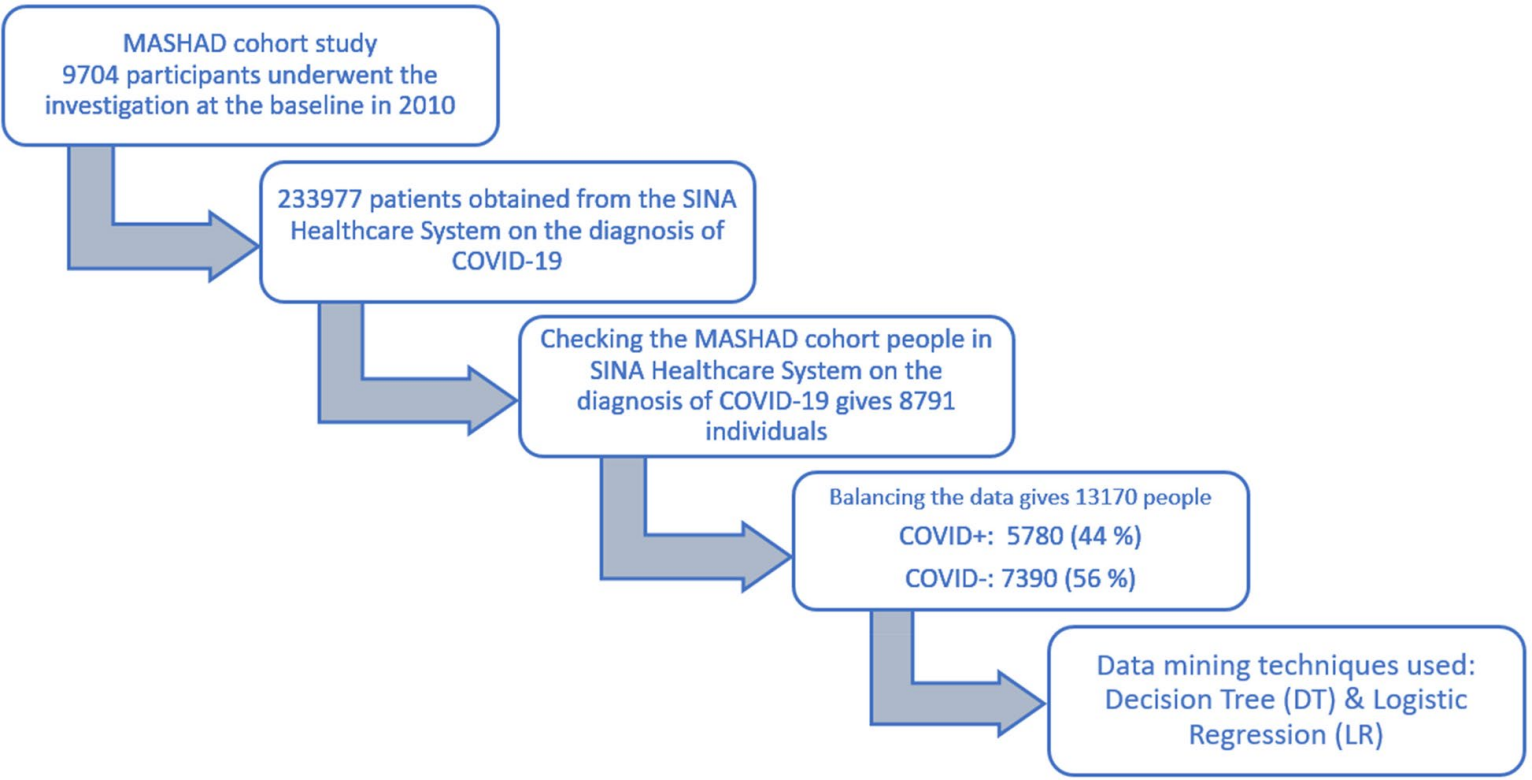
Flow chart of this study

Data on the diagnosis of COVID-19 was obtained from the SINA Healthcare System, which records the electronic health profiles of patients in hospitals and health centers in Mashhad, Iran. Data collection began from the onset of the disease to the end of March 2021. Diagnosis of the disease was confirmed using a lung spiral computerized tomography (CT) scan and/or polymerase chain reaction (PCR) laboratory test. The flow chart of this study is given in Fig. 1.

### Statistical analysis and model building

For analyzing the data, SAS JMP Pro version 13 (SAS Institute Inc., Cary, NC) and SPSS version 22 (Armonk, NY: IBM Corp.) were applied. Chi-square and Fisher’s exact tests were applied to measure the association between categorical variables. Also, T independent test is for comparing the means not for normality.

In this study there was an unbalanced dataset (Cov + compared to Cov-). Thus, a Synthetic Minority Oversampling Technique (SMOTE) algorithm was used in LR, DT, and BF algorithms to transform the unbalanced data set into a balanced one [21, 22]. Based on SMOTE algorithm, sampling was done from 10 observations so that 8 or 9 cases of disease and a maximum of 2 cases of non-disease were selected. In each step, the samples were repeated based on the posterior distribution function. These steps were continued until the number of cases of the disease was very close to another category, i.e., non-infection.

LR is a statistical model, which is utilized to model dichotomous targets and deducing the effect of explanatory variables on the dichotomous target variable [23, 24]. Providing a good direct or inverse association between the inputs or explanatory variables and the target is the main advantage of applying LR algorithm.

In order to evaluate the performance of the LR, DT, and BF algorithms and comparisons, we gave the confusion matrix (Accuracy, Sensitivity, Precision, and Area Under Curve (AUC) of the receiver operating characteristics (ROC) curve) of the algorithms for training data and also for all models.

## Results

A total of 13,170 participants were recruited (*n* = 5780 people infected to SARS-COV-2 (case) and *n* = 7390 individuals without SARS-COV-2 (control)). Based on Table 1, participants with SARS-COV-2 were significantly older than the control group (59.29 ± 8.54 versus 56.97 ± 9.03 years, respectively). In addition, BMI, diastolic blood pressure (DBP), systolic blood pressure (SBP), blood urea nitrogen (BUN), sex, smoking status, serum zinc, copper, creatinine (Cr), cholesterol, triglyceride, high sensitivity C-Reactive Protein (hs-CRP), fasting blood glucose (FBG), serum phosphorus, low-density lipoprotein cholesterol (LDL-C), high-density lipoprotein cholesterol (HDL-C), serum gamma glutamyl transferase (Gamma-GT), creatine phosphokinase (CPK), serum calcium, serum total bilirubin, serum direct bilirubin, aspartate aminotransferase (AST), alanine transaminase (ALT), alkaline phosphatase (ALP), serum uric acid and magnesium showed significant differences between groups. Several hematological factors, white blood cells (WBC), red blood cells (RBC), hemoglobin, hematocrit, mean corpuscular volume (MCV), mean corpuscular hemoglobin (MCH), mean corpuscular hemoglobin concentration (MCHC), red cell distribution width (RDW), platelet distribution width (PDW), and mean platelet volume (MPV) were higher compared to the control group (*P*-value < 0.05).

**Table 1:** Summary of the demographic characteristics of this study

**Variables**		Cov+ (5780)	Cov– (7390)	*P*-Value
		58.80±9.63	57.09±8.77	<0.001
**Gender n (%)**	**Female**	2500 (43.3)	4667 (63.3)	<0.001
	**Male**	3276 (56.7)	2704 (36.7)	
**Smoking status n (%)**	**Non smoker**	369(77.8)	5418(74.2)	<0.001††
	**Ex–smoker**	50(10.5)	527(7.2)	
	**Current smoker**	55(11.6)	1350(18.5)	
**BMI (Kg/m**^**2**^**)**		28.56±4.58	28.36±4.88	0.026
**SBP (mmHg)**		135.90±21.11	134.84±20.75	<0.001
**DBP (mmHg)**		81.62±14.91	81.78±13.92	<0.001
**Serum Zinc (mg/dl)**		85.44±19.58	85.35±28.05	0.513†
**Serum Copper (mg/dl)**		105.13±37.43	103.99±38.20	0.582†
**Serum Cr (mg/dl)**		1.25±0.31	1.10±0.23	<0.001†
**Serum BUN (mg/dl)**		34.80±10.51	33.20±10.09	0.007
**Serum Cholesterol (mg/dl)**		200.36±47.50	205.89±44.69	<0.001
**Serum Triglyceride (mg/dl)**		149.35±88.41	147.36±80.36	<0.001
**Serum Calcium (mg/dl)**		9.66±0.52	9.67±0.58	0.021
**FBG (mg/dl)**		118.91±49.45	113.66±43.67	<0.001†
**Serum hs-CRP (mg/l)**		2.91±4.02	2.82±4.46	0.031†
**Serum Phosphorus (mg/dl)**		3.91±0.46	3.90±0.46	<0.001†
**Serum HDL-C (mg/dl)**		48.17±10.96	48.79±10.73	<0.001†
**Serum LDL-C (mg/dl)**		113.04±41.69	116.52±35.11	<0.001†
**Serum AST (mg/dl)**		22.55±9.76	22.10±9.22	<0.001
**Serum ALT (mg/dl)**		19.71±12.58	19.19±12.82	<0.001
**Serum ALP (IU/l)**		220.72±71.46	223.87±67.81	<0.001
**Serum Gamma-GT (IU/l)**		28.63±29.76	25.77±23.25	0.024†
**Serum CPK (IU/l)**		124.67±82.35	120.75±80.72	0.006†
**Serum Direct Bilirubin (mg/dl)**		0.25±0.10	0.25±0.13	<0.001
**Serum Total Bilirubin (mg/dl)**		0.86±0.36	0.83±0.32	<0.001†
**Serum Iron (mcg/dl)**		90.84±35.92	91.85±36.59	<0.001
**Serum Magnesium (mg/dl)**		2.32±0.25	2.35±0.25	<0.001†
**Serum Uric Acid (mg/dl)**		5.22±1.29	5.05±1.33	0.006
**Hematologic parameters**	**WBC (×10**^**3**^**/µl)**	6.26±1.61	6.38±2.02	<0.001†
	**RBC (×10**^**3**^**/µl)**	4.87±0.52	4.86±0.48	<0.001
	**Hemoglobin (g/dl)**	14.35±1.60	14.31±1.55	<0.001
	**Hematocrit (%)**	41.67±3.99	41.65±3.90	<0.001
	**MCV (fl)**	85.76±5.86	85.80±5.89	<0.001†
	**MCH (pg)**	29.53±2.41	29.47±2.49	<0.001†
	**MCHC (g/dl)**	34.43±1.62	34.33±1.69	<0.001
	**Platelets (×10**^**3**^**/µl)**	238.05±58.08	242.78±62.43	<0.001
	**RDW (%)**	13.26±1.07	13.24±1.17	<0.001†
	**PDW (%)**	12.52±2.02	12.69±2.16	<0.001†
	**MPV (fl)**	10.12±0.94	10.02±0.96	<0.001†

Two independent T-test was used, †Mann–Whitney U tests, ††Chi-Square test

*Abbreviations*: *LDL-C* Low density lipoprotein cholesterol, *HDL-C* High density lipoprotein cholesterol, *hs-CRP* High-sensetive C reactive proptein, *AST* Aspartate aminotransferase, *ALT*: Alanine aminotransferase, *Cr* Creatinine, *BMI* Body mass index, *DBP* Diastolic blood pressure, *SBP* Systolic blood pressure, *BUN* Blood urea nitrogen, *FBG* Fasting blood glucose, *Gamma-GT* Gamma glutamyl transferase, *CPK* Creatine phosphokinase, *ALP* Alkaline phosphatase, *WBC* White blood cells, *RBC* Red blood cells, *MCV* Mean corpuscular volume, *MCH* Mean corpuscular hemoglobin, *MCHC* Mean corpuscular hemoglobin concentration, *RDW* Red cell distribution width, *PDW* Platelet distribution width, *MPV* Mean platelet volume

### Main findings

We have attempted to use the LR, DT, and BF models to diagnostic COVID-19 tested participants and their biochemical and hematologic features. In this regard, the data were divided into two parts as training and test data (80%-20%), randomly. The models are validated using test data (20%) and built on the training dataset. Results of the LR algorithm illustrated that biochemical factors (Model I), such as age, smoking status, sex, DBP, SBP, BUN, BMI, hs-CRP, FBG, HDL-C, AST, ALT, CPK, total bilirubin, iron, magnesium, and Gamma-GT were correlated with COVID-19 status (*P*-value < 0.05). In Model I, the BMI, BUN, age variables have been defined as the most crucial variable with high OR by the LR algorithm. With a unit increase in BMI, the chance of being Cov + was 1.092 times. With a year increase in age, the chance of being Cov + was 1.048 times, and with a unit increase in BUN, the chance of being Cov + was 1.041 (see Table 2). In Model II, BMI, age, hemoglobin, hematocrit, sex, MPV, smoking status, and MCHC were significant (*P*-value < 0.05). The hemoglobin had an OR equal to 4.292, so, the chance of being Cov + was 4.292 times. The MPV had an OR equal to 1.550, so, the chance of being Cov + was 1.550 times. Table 3 showed the other variables and values of effect. In Model III, CPK, BMI, MPV, FBG, sex, BUN, Cr, iron, magnesium, total bilirubin, hemoglobin, hematocrit, MCHC, smoking status, age, WBC, HDL-C, and ALT were correlated with COVID-19 status (*P*-value < 0.05). The total bilirubin and MPV had an OR 1.647 and 1.447, so, the chance of being Cov + was 1.647 and 1.447 times, respectively (see Table 4). Based on Table 5, for LR algorithm the accuracy of three models (Model I, II, and III) were 75.13%, 68.28%, and 69.63%, respectively. The other performance indices were given in Table 5 (a), (d), and (g).

**Table 2:** The results of LR algorithms for Model I

Variables	Log-Worth	OR (95% CI)	S. E	*P*-Value*
CPK	54.576	1.006 (1.005, 1.007)	< 0.001	< 0.001
SBP	36.776	1.036 (1.030, 1.042)	0.002	< 0.001
BMI	34.485	1.092 (1.076, 1.107)	0.007	< 0.001
BUN	34.262	1.041 (1.034, 1.047)	0.003	< 0.001
DBP	32.548	1.048 (1.041, 1.057)	0.004	< 0.001
Age	26.749	1.048 (1.040, 1.058)	0.004	< 0.001
FBG	17.254	1.007 (1.005, 1.008)	< 0.001	< 0.001
Sex [female]	16.156	0.576 (0.506, 0.656)	0.033	< 0.001
Total Bilirubin	13.189	0.500 (0.416, 0.601)	0.093	< 0.001
Iron	8.595	1.006 (1.004, 1.008)	< 0.001	< 0.001
Magnesium	7.256	0.408 (0.295, 0.565)	0.165	< 0.001
Smoking status [no]	6.140	1.134 (0.975, 1.320)	0.052	< 0.001
Smoking status [current]	6.140	0.881 (0.758, 1.026)	0.062	0.0308
HDL-C	3.924	0.987 (0.980, 0.994)	0.003	< 0.001
ALT	3.815	0.983 (0.974, 0.992)	0.004	< 0.001
hs-CRP	3.691	0.970 0.955, 0.986)	0.008	< 0.001
Gamma-GT	3.458	1.007 (1.003, 1.010)	0.001	< 0.001
AST	2.252	1.018 (1.005, 1.031)	0.006	0.005

^*^Significant at error level 0.05

*Abbreviations:* *HDL-C* High density lipoprotein cholesterol, *hs-CRP* High-sensetive C reactive proptein, *AST* Aspartate aminotransferase, *ALT* Alanine aminotransferase, *BMI* Body mass index, *DBP* Diastolic blood pressure, *SBP* Systolic blood pressure, *BUN* Blood urea nitrogen, *FBG* Fasting blood glucose, *Gamma-GT* Gamma glutamyl transferase, *CPK* Creatine phosphokinase

**Table 3:** The results of LR algorithms for Model II

Variables	Log-Worth	OR (95% CI)	S. E	*P*-Value*
Hemoglobin	5.188	4.292 (2.238, 8.455)	0.339	< .001
Hematocrit	5.003	0.614 (0.487, 0.767)	0.116	< .001
MCHC	3.788	0.598 (0.451, 0.786)	0.142	< .001
MPV	66.236	1.550 (1.475, 1.633)	0.026	< .001
Sex[female]	93.749	0.337 (0.303, 0.374)	0.027	< .001
Age	59.774	1.048 (1.043, 1.055)	0.003	< .001
Smoking status [no]	9.034	1.852 (1.530, 2.242)	0.040	< .001
Smoking status [current]	9.034	0.591 (0.479, 0.731)	0.049	0.002
BMI	99.923	1.120 (1.107, 1.131)	0.001	< .001

^*^ Significant at error level 0.05

*Abbreviations*: *MCHC* Mean corpuscular hemoglobin concentration, *MPV*: Mean platelet volume, *BMI* Body mass index

**Table 4:** The results of LR algorithms for Model III

Variables	Log-Worth	OR (95% CI)	S. E	*P*-Value*
WBC	4.128	1.081 (1.040, 1.123)	0.019	< .001
Hemoglobin	7.858	9.534 (4.216, 22.291)	0.425	< .001
Hematocrit	7.579	0.469 (0.350, 0.620)	0.145	< .001
MCHC	6.139	0.440 (0.310, 0.617)	0.176	< .001
MPV	36.887	1.447 (1.366, 1.531)	0.029	< .001
Sex[female]	19.381	0.551 (0.485, 0.626)	0.032	< .001
Age	4.719	1.015 (1.008, 1.022)	0.003	< .001
Smoking status [no]	5.985	1.737 (1.400, 2.156)	0.046	< .001
Smoking status [current]	5.985	0.662 (0.520, 0.844)	0.056	< .001
BMI	42.593	1.087 (1.074, 1.101)	0.006	< .001
Cr	10.158	0.376 (0.279, 0.505)	0.151	< .001
BUN	17.925	1.030 (1.024, 1.038)	0.003	< .001
FBG	19.787	1.006 (1.005, 1.007)	< .001	< .001
HDL	3.747	0.989 (0.983, 0.995)	0.003	< .001
ALT	3.092	1.008 (1.003, 1.013)	0.002	< .001
CPK	66.551	1.006 (1.005, 1.007)	< .001	< .001
Total Bilirubin	8.284	1.647 (1.393, 1.949)	0.085	< .001
Iron	9.906	1.006 (1.004, 1.008)	< .001	< .001
Magnesium	9.002	0.415 (0.313, 0.550)	0.143	< .001

^*^ Significant at error level 0.05

*Abbreviations:* *ALT* Alanine aminotransferase, *Cr* Creatinine, *BMI* Body mass index, *BUN* Blood urea nitrogen, *FBG* Fasting blood glucose, *CPK* Creatine phosphokinase, *WBC* White blood cells, *MCHC* Mean corpuscular hemoglobin concentration, *MPV* Mean platelet volume

**Table 5:** Model performance indices of the LR, DT, BF algorithms for Model I, II, and III in training data

Model I						
**(a) LR**				**(b) DT**		
**Actual**	**Predicted Count**			**Actual**	**Predicted Count**	
**COVID Positive**	**No**	**Yes**		**COVID Positive**	**No**	**Yes**
**No**	3328	675		**No**	5149	758
**Yes**	1075	1959		**Yes**	2061	2568
Sensitivity = 83.14%	AUC = 80.74%			Sensitivity = 87.17%	AUC = 80.23%	
Precision = 75.58%	Accuracy = 75.13%			Precision = 71.41%	Accuracy = 73.24%	
**(c) BF**						
**Actual**	**Predicted Count**					
**COVID Positive**	**No**		**Yes**			
**No**	5718		189			
**Yes**	819		3810			
Sensitivity = 96.80 %			AUC = 98.06 %			
Precision = 87.47 %			Accuracy = 90.43 %			
**Model II**						
**(d) LR**				**(e) DT**		
**Actual**	**Predicted Count**			**Actual**	**Predicted Count**	
**COVID Positive**	**No**		**Yes**	**COVID Positive**	**No**	**Yes**
**No**	4074		1175	**No**	4506	1401
**Yes**	1764		1175	**Yes**	1401	2925
Sensitivity = 77.61 %			AUC = 77.37 %	Sensitivity = 76.28 %		Sensitivity = 76.28 %
Precision = 69.78 %			Accuracy = 68.28 %	Sensitivity = 76.28 %		Sensitivity = 76.28 %
**(f) BF**						
**Actual**	**Predicted Count**					
**COVID Positive**	**No**		**Yes**			
**No**	5488		419			
**Yes**	1262		3367			
Sensitivity = 92.91 %			Precision = 81.30 %			
Precision = 81.30 %			Accuracy = 84.05 %			
**Model III**						
**(g) LR**				**(h)DT**		
**Actual**	**Predicted Count**			**Actual**	**Predicted Count**	
**COVID Positive**	**No**		**Yes**	**Predicted Count**	**No**	**No**
**No**	3890		871	**No**	5282	625
**Yes**	1273		2427	**Yes**	2176	2453
Sensitivity = 66.08%			AUC = 80.37 %	Sensitivity = 66.00%		Precision = 72.88%
Precision = 74.93%			AUC = 80.37 %	Precision = 72.88%		Precision = 72.88%
**(i) BF**						
**Actual**	**Predicted Count**					
**COVID Positive**	**No**		**Yes**			
**No**	5808		99			
**Yes**	647		3982			
Sensitivity = 66.08%			AUC = 99.00 %			
Precision = 74.93%			Accuracy = 69.63%			

In the training phase of DT, the important variables were selected and the final tree is given after pruning. Models I, II, and III runs with 17, 8, and 18 variables as input, respectively. In Model I, CPK, age, BUN, BMI, ALP, sex, total bilirubin, hs-CRP, FBG, and Gamma-GT, in Model II, age, MPV, sex, BMI, hemoglobin, and MCHC, and in Model III, CPK, Cr, BUN, BMI, FBG, age, MPV, MCHC, sex, and total bilirubin variables remained in models. Based on Table 5, the tree is made based on biochemical, hematologic, and both of the variables (Model I, Model II, and Model III, respectively) that had 73.24%, 70.53%, and 68.80% accuracy on the training data, respectively. The other performance indices were given in Table 5 (b), (e), and (h).

The rules from DTs for Model I, II, and III is shown in Table 6. Rule 1 in Model I was illustrated that in a subgroup with CPK >  = 114.09 & BUN >  = 30.00 & BMI >  = 26.77 & Age >  = 54.00 & Gamma-GT >  = 16.91, the chance or probability of having Cov + was 84.69%. In another subgroup, CPK < 114.09 & CPK < 88.06 & Sex(female) & ALT < 9.00 led to a 6.57% chance of having Cov + . The rules from Model II, were illustrated that there was an 86.46% chance that participants with features such as Age >  = 54.00 & BMI >  = 26.77 & MPV >  = 9.60 & Sex(male) & Hemoglobin < 15.8 be infected with COVID-19. Another rule was suggested that the probability of Cov + in individuals with Age < 54.00 & MPV < 9.10 was 12.26%. The rules from Model III, were illustrated that there was an 88.15% chance that participants with features such as CPK >  = 114.09 & BUN >  = 30.00 & BMI >  = 26.77 & Age >  = 54.00 & MPV >  = 9.60 & MCHC < 35.6 be infected with COVID-19. Another rule was suggested that the probability of Cov + in individuals with CPK < 114.09 & Cr < 1.40 & Cr < 1.00 & FBG < 118.34 & Sex(female) was 9.90%. Other rules were stated in Table 6.

**Table 6:** Extracted rules the DT algorithms for Model I, II, and III

Model I			
**Num**	**Rules**	**Cov- (%)**	**Cov + (%)**
1	CPK > = 114.09 & BUN > = 30.00 & BMI > = 26.77 & Age > = 54.00 & Gamma-GT > = 16.91	15.31	84.69
2	CPK > = 114.09 & BUN > = 30.00 & BMI > = 26.77 & Age > = 54.00 & Gamma-GT < 16.91	53.73	46.27
3	CPK > = 114.09 & BUN > = 30.00 & BMI > = 26.77 & Age < 54.00	53.99	46.01
4	CPK > = 114.09 & BUN > = 30.00 & BMI < 26.77 & FBG > = 121.38	36.13	63.87
5	CPK > = 114.09 & BUN > = 30.00 & BMI < 26.77 & FBG < 121.38	73.70	26.30
6	CPK > = 114.09 & BUN < 30.00 & FBG > = 124.01	48.98	51.02
7	CPK > = 114.09 & BUN < 30.00 & FBG < 124.01 & Total Bilirubin > = 0.72	71.80	28.20
8	CPK > = 114.09 & BUN < 30.00 & FBG < 124.01 & Total Bilirubin < 0.72	89.61	10.39
9	CPK < 114.09 & CPK > = 88.06 & BUN > = 38.13	47.25	52.75
10	CPK < 114.09 & CPK > = 88.06 & BUN < 38.13 & hs-CRP > = 0.62 & BUN > = 26.05	65.61	34.39
11	CPK < 114.09 & CPK > = 88.06 & BUN < 38.13 & hs-CRP > = 0.62 & BUN < 26.05	83.86	16.14
12	CPK < 114.09 & CPK > = 88.06 & BUN < 38.13 & hs-CRP < 0.62	78.79	21.21
13	CPK < 114.09 & CPK < 88.06 & Sex(male)	69.06	30.94
14	CPK < 114.09 & CPK < 88.06 & Sex(female) & ALT > = 9.00 & Total Bilirubin > = 0.80	75.92	24.08
15	CPK < 114.09 & CPK < 88.06 & Sex(female) & ALT > = 9.00 & Total Bilirubin < 0.80	85.98	14.02
16	CPK < 114.09 & CPK < 88.06 & Sex(female) & ALT < 9.00	93.43	6.57
**Model II**			
**Num**	**Rules**	**Cov- (%)**	**Cov + (%)**
1	Age > = 54.00 & BMI > = 26.77 & MPV > = 9.60 & Sex(male) & Hemoglobin < 15.8	13.54	86.46
2	Age > = 54.00 & BMI > = 26.77 & MPV > = 9.60 & Sex(male) & Hemoglobin > = 15.8	47.74	52.26
3	Age > = 54.00 & BMI > = 26.77 & MPV > = 9.60 & Sex(female)	44.60	55.40
4	Age > = 54.00 & BMI > = 26.77 & MPV < 9.60	65.00	35.00
5	Age > = 54.00 & BMI < 26.77 & Age > = 59.04	67.60	32.40
6	Age > = 54.00 & BMI < 26.77 & Age < 59.04	81.82	18.18
7	Age < 54.00 & MPV > = 9.10 & MCHC > = 32.31	70.35	29.65
8	Age < 54.00 & MPV > = 9.10 & MCHC < 32.31	91.70	8.30
9	Age < 54.00 & MPV < 9.10	87.74	12.26
**Model III**			
**Num**	**Rules**	**Cov- (%)**	**Cov + (%)**
1	CPK > = 114.09 & BUN > = 30.00 & BMI > = 26.77 & Age > = 54.00 & MPV > = 9.60 & MCHC < 35.6	11.85	88.15
2	CPK > = 114.09 & BUN > = 30.00 & BMI > = 26.77 & Age > = 54.00 & MPV > = 9.60 & MCHC > = 35.6	57.48	42.52
3	CPK > = 114.09 & BUN > = 30.00 & BMI > = 26.77 & Age > = 54.00 & MPV < 9.60	46.60	53.40
4	CPK > = 114.09 & BUN > = 30.00 & BMI > = 26.77 & Age < 54.00	55.05	44.95
5	CPK > = 114.09 & BUN > = 30.00 & BMI < 26.77	64.34	35.66
6	CPK > = 114.09 & BUN < 30.00 & FBG > = 139.05	40.88	59.12
7	CPK > = 114.09 & BUN < 30.00 & FBG < 139.05 & Total Bilirubin > = 0.72	70.06	29.94
8	CPK > = 114.09 & BUN < 30.00 & FBG < 139.05 & Total Bilirubin < 0.72	88.82	11.18
9	CPK < 114.09 & Cr > = 1.40	36.39	63.61
10	CPK < 114.09 & Cr < 1.40 & Cr > = 1.00	70.76	29.24
11	CPK < 114.09 & Cr < 1.40 & Cr < 1.00 & FBG > = 118.34	73.48	26.52
12	CPK < 114.09 & Cr < 1.40 & Cr < 1.00 & FBG < 118.34 & Sex(male)	80.97	19.03
13	CPK < 114.09 & Cr < 1.40 & Cr < 1.00 & FBG < 118.34 & Sex(female)	90.10	9.90

*Abbreviations:* *hs-CRP* high-sensetive C reactive proptein, *ALT* Alanine aminotransferase, *Cr* Creatinine, *BMI* body mass index, *BUN* Blood urea nitrogen, *FBG* Fasting blood glucose, *Gamma-GT* Gamma glutamyl transferase, *CPK *Creatine phosphokinase, *MCV* Mean corpuscular volume, *MCHC* Mean corpuscular hemoglobin concentration, *MPV* Mean platelet volume, *Num* Number of rules

**Fig. 2:**
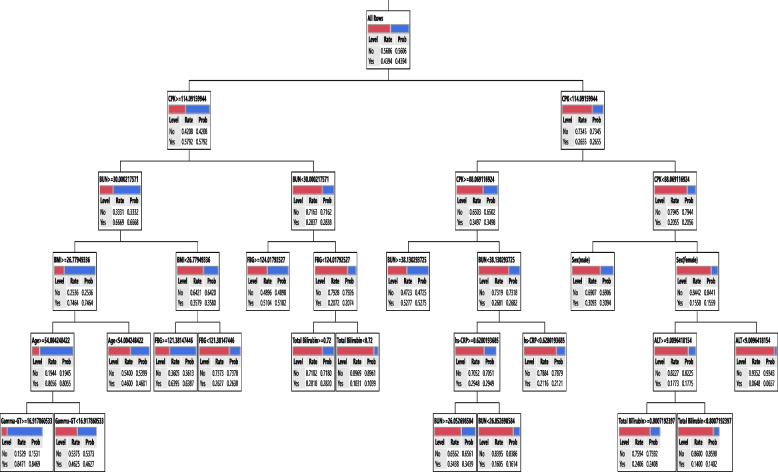
Graphical representation of the classification tree introduced for SARS-COV-2 diagnosis for Model I

**Fig. 3:**
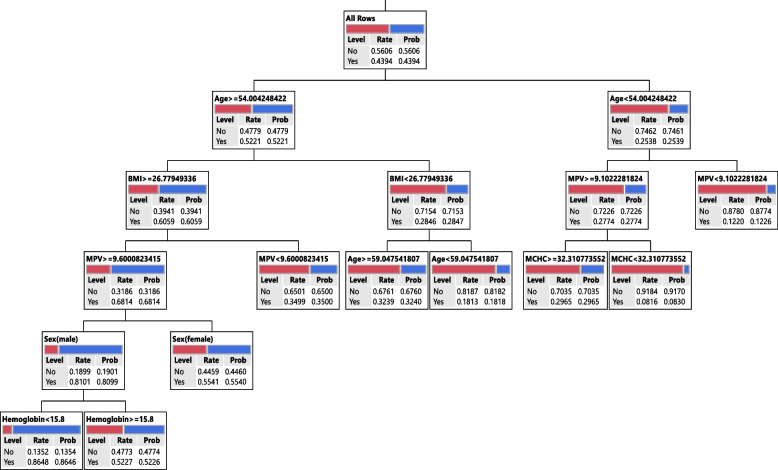
Graphical representation of the classification tree introduced for SARS-COV-2 diagnosis for Model II

**Fig. 4:**
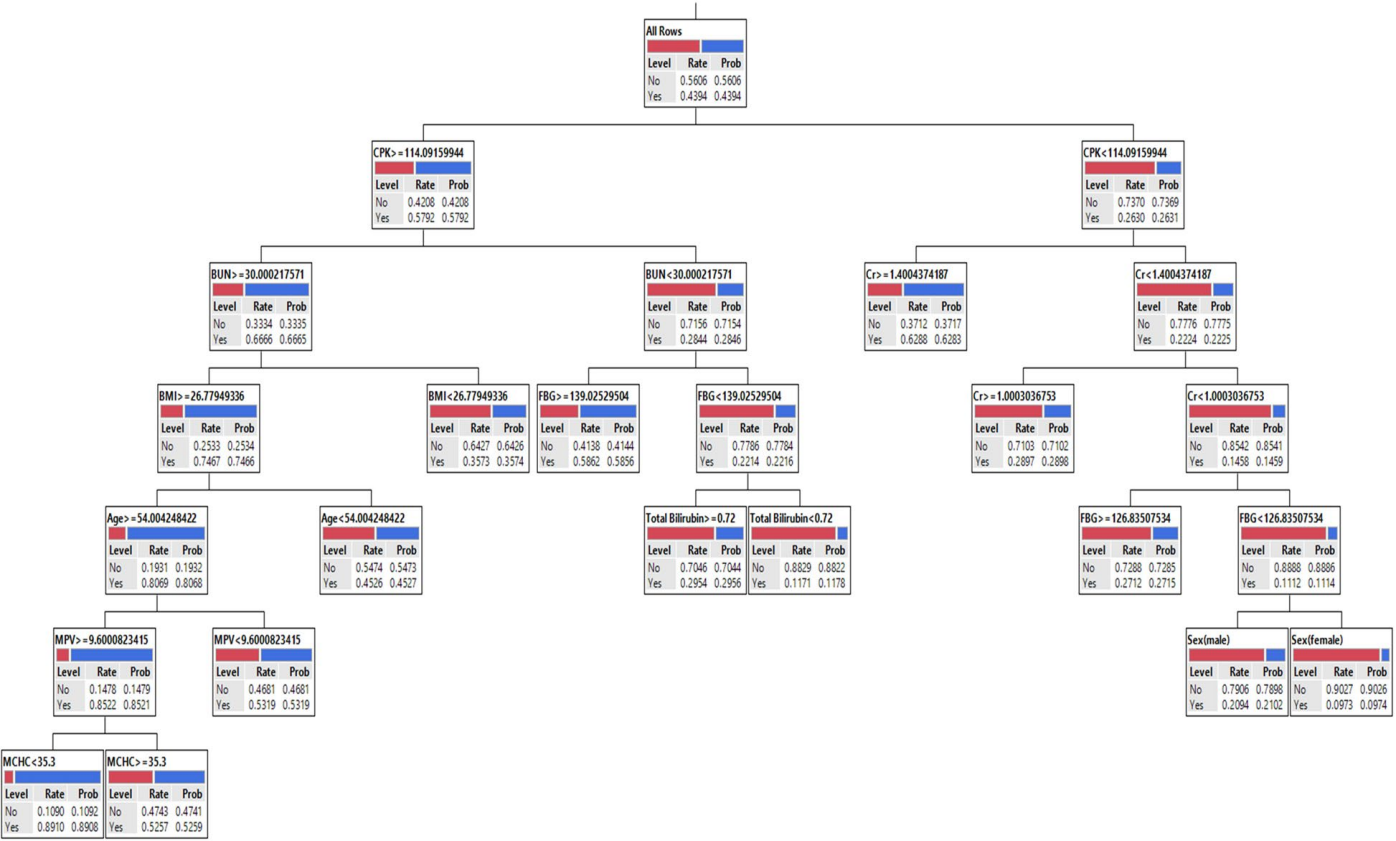
Graphical representation of the classification tree introduced for SARS-COV-2 diagnosis for Model III

Hence, the CPK and BUN for Model I, age, BMI, and MPV for Model II, and CPK and BUN for Model III were defined as most crucial variables. The final DT is shown in Figs. 2, 3, and 4.

In the final step, for another analysis we applied BF for analyzing the data based on COVID-19. The factors included in the BF algorithm were 17, 8, and 18 variables for Model I, II, and III, respectively. Moreover, we set the following specifications for Model I: Number of Trees in the Forest: 29 for Model I, 13 for Model II, and 53 for Model III, Number of Terms Sampled per Split: 4 for Model I, 2 for Model II, and 4 for Model III, Training Rows: 10,536, Test Rows: 2634, Minimum Splits per Tree: 10, Minimum Size Split: 13 for all three models. Confusion matrix and evaluation indices for comparison of the models I, II, III were stated in Table 5 (c), (f), and (i). Additionally, the crucial variables related to COVID-19 based on BF algorithm were: CPK, BUN, FBG, BMI, total bilirubin, and age in Model I, BMI, sex, MPV, and age in Model II, and CPK, Cr, FBG, BMI, BUN, total bilirubin, sex, MPV, and age for Model III. As one can check the obtained features from BF algorithm were equal to the obtained factors from LR and DT algorithms.

## Discussion

This cohort and retrospective study which compared 5780 infected participants to COVID-19 and 7390 subjects without COVID-19 from Mashhad, Iran in terms of baseline profiles, clinical features, and outcomes. We investigated the relationship between sex, age, BMI, SBP, DBP, and smoking status as demographical factors, biochemical features including BUN, serum zinc, copper, Cr, triglyceride, cholesterol, FBG, hs-CRP, phosphorus, LDL-C, HDL-C, Gamma-GT, CPK, direct bilirubin, calcium, total bilirubin, AST, ALT, ALP, uric acid, and magnesium, and hematologic features including WBC, RBC, hemoglobin, hematocrit, MCV, MCH, MCHC, RDW, PDW, and MPV with COVID-19 through DT, BF, and LR algorithms, to obtain the related parameters and the best predicting factors. We propose three models, in Model I, the association between COVID-19 and biochemical features, in Model II, the association between COVID-19 and hematologic features, and in Model III, the association between COVID-19 and both biochemical and hematologic features were assessed. In Model I, our BF, DT, and LR algorithms illustrated that CPK, BUN, FBG, BMI, total bilirubin, sex, and age, as important predictors. In Model II, our BF, DT, and LR algorithms illustrated that BMI, sex, MPV, and age as important predictors. Finally, in Model III, our BF, DT, and LR algorithms illustrated that CPK, BMI, MPV, BUN, FBG, sex, Cr, age, and total bilirubin as important predictors.

This paper attempts to show that graphical representation of the classification tree for hematologic factors (Model II). The DT with 5 layers, identified the various risk factors for SARS-COV-2. Based on our results, in the subgroup with Age >  = 54, BMI ≥ 26.7, MPV ≥ 9.6, and hemoglobin < 15.8, eighty-six percent of subjects were classified in the patient group. Also, in a subgroup of individuals with Age < 54, MPV ≥ 9.1, and MCHC ≥ 32.2 < 35.3, 29% of individuals were in the patient group. Since hematological factors appeared as the first factors in the DT, these results match those observed in earlier studies. Some authors have indicated that the involvement of the hematopoietic system is associated with severe cases and also with poor outcomes and mortality. Para clinic abnormalities including Lymphopenia, thrombocytopenia, leukopenia, and a prothrombotic state are public manifestations of COVID-19 [25]. The finding of Jalil et al. (2022) on hematological and serological parameters for detection of COVID-19 showed that the levels of hematocrit, MCV, MCH, Pelt, WBC, LYM, Mid, MPV, PCT decreased, but level of hemoglobin, RBC, GRAN% increase in patient with COVID-19 [26]. It suggested that hematological parameters have important role in prognostic implications.

SARS-COV-2 has a high transmission potential, especially in the elderly and those with underlying diseases [7]. Numerous studies have attempted to show the COVID-19 incidence in people with metabolic disorders, especially diabetics who are prone to COVID-19 due to a compromised immune system [27–29]. Diabetes is one of the most frequent underlying comorbidities in patients with COVID-19, according to recent reports, and it is related to prevalence and mortality in these patients [30, 31]. The present study makes several noteworthy contributions to the critical feature of the relationship between demographic, biochemical, and hematological characteristics, in patients with and without COVID-19 infection by data mining approaches. In the same vein, a data mining study by Marhl et al. aimed to deduce the physiological roots of clinical findings relating diabetes to the severity and adverse effect of SARS-COV-2. They also suggested clinical biomarkers that could predict a higher risk, such as HTN, elevated serum alanine aminotransferase, high Interleukin-6, and a low lymphocyte count [32–34].

The results of some studies consistently indicated a high incidence of diabetes in SARS-COV-2 patients (24.9%) and statistically significant statistical difference between SARS-COV-2 patients with diabetes and those without diabetes in hospitalized SARS-COV-2 patients [31, 35]. The most striking result to emerge from the data is that that serum levels of FBG were significantly different between case and control groups. Also, as DT and BF showed, serum levels of FBG were significantly increase the risk of COVID-19.

Furthermore, there was a significant difference in LDL-C levels between the case and control groups. Similarly, Wei et al. found that LDL-C levels in SARS-COV-2 patients were slightly lower than in healthy participants [36].

According to data from China, while men and women have the same prevalence of SARS-COV-2, infected men were more likely to die than women [37, 38]. Here, all models illustrated that the incidence of COVID-19 was more in men.

There was an association between smoking and COVID-19, which was in country with a recent meta-analysis study [39–41]. In fact, the obtained results showed that, the incidence of COVID-19 was more in smokers.

In our LR algorithm in Model I, a significant correlation was found in SBP and DBP with COVID-19 which increased the incidence. In accordance with the results from Schiffrin et al. (2020), it is uncertain whether uncontrolled HTN is a risk factor for SARS-COV-2 infection [42] while, Pranata et al. investigated that HTN was a high risk of death, severe COVID-19, acute respiratory distress syndrome (ARDS), intensive care unit (ICU) admission, and disease progression in COVID-19 patients [43]. High SBP is a source of end-organ damage and a significant comorbid factor, according to a new report published in 2021 [44].

In this study, we identified an association between SARS-COV-2 and component factors of dyslipidemia such as cholesterol, triglycerides, and HDL-C. In fact, LR algorithm showed that HDL-C decreased the incidence of infection. As stated by Hariyanto et al., dyslipidemia increases the risk of experiencing serious outcomes from SARS-COV-2 infections [45]. In 2020, several studies investigated to describe the correlation of lipid profile and COVID-19. Hua et al. found that serum HDL-C concentrations decreased significantly in the early stages of SARS-COV-2 infection [46] and Wei Ye et al. have found a substantial decrease in cholesterol levels in COVID-19 patients' serum [36]. This result may be explained by the fact that HDL-C, LDL-C, Triglyceride, and Cholesterol level in the baseline of our study is significant between the studied groups.

Based on the findings from Zhu et al., the positive chest CT scan of COVID-19 patients were correlated with CRP levels which showed that CRP levels rise in the majority of serious and critical cases, and were associated to their prognosis [47]. By the way, there was a relationship between hs-CRP levels and SARS-COV-2 in this study.

In accordance with the published results, hospitalized patients with COVID-19 infection had impaired liver function. Their liver inflammatory markers including AST, ALT, ALP, total bilirubin, and Gamma-GT have been elevated [48–50]. The obtained results of this study in majority cases confirm the previous research.

Electrolyte balance and adequate mineral and vitamin intake are main parameters that impact disease progression. Since they have an effect on the immune system, electrolyte imbalance and lack of trace elements or vitamins raise the risk of serious infection [51]. Iron, magnesium, uric acid, calcium, and BUN were investigated in current research, and it was found that they had an association with SARS-COV-2.

A limitation of this study is that the numbers of patients were relatively small. The current research was not specifically designed to evaluate anthropometric parameters and nutritional questionnaires. It is suggested that the association of these factors is investigated in future studies.

## Conclusion

This project was undertaken to design and evaluate biochemical and hematological assessment in the MASHAD cohort study and compare these between COVID-19 infected patients and non-infected subjects. Our DT and BF model appears to be able to predict and classify infected and non-infected people based on biochemical and hematologic factors which had an association with SARS-COV-2.

## Data Availability

The datasets used and/or analyzed during the current study available from the corresponding author on reasonable request.

## Abbreviations

COVID-19: Coronavirus Disease 2019
SARS: Severe Acute Respiratory Syndrome
OR: Odds Ratios
LR: Logistic Regression
DT: Decision Tree
BF: Bootstrap Forest
AUC: Area Under Curve
ROC: Receiver Operating Characteristics
FBG: Fasting Blood Glucose
SBP: Systolic Blood Pressure
DBP: Diastolic Blood Pressure
WBC: White Blood Cell
RBC: Red Blood Cell
MCV: Mean Corpuscular Volume
MCH: Mean Corpuscular Hemoglobin
MCHC: Mean Corpuscular Hemoglobin Concentration
RDW: Red Cell Distribution
PDW: Platelet Distribution Width
MPV: Mean Platelet Volume
BUN: Blood Urea Nitrogen
CPK: Creatine Phosphokinase
BMI: Body Mass Index
hs-CRP: High Sensitivity C-Reactive Protein
Cr: Creatinine
ALP: Alkaline Phosphatase
Gamma-GT: Gamma Glutamyl Transferase
LDL-C: Low-Density Lipoprotein Cholesterol
HDL-C: High-Density Lipoprotein Cholesterol
AST: Aspartate Aminotransferase
ALT: Alanine Transaminase
LDH: Lactate Dehydrogenase
ARDS: Acute Respiratory Distress Syndrome
ICU: Intensive Care Unit
CT: Computerized Tomography
PCR: Polymerase Chain Reaction
